# Injection granuloma mimicking soft tissue sarcoma following seasonal influenza vaccine administration: A case report

**DOI:** 10.1097/MD.0000000000028942

**Published:** 2022-04-22

**Authors:** Gregory R. Toci, Christa L. LiBrizzi, Jarred A. Bressner, Adam Scott Levin, Carol D. Morris

**Affiliations:** a The Johns Hopkins University School of Medicine, Baltimore, MD; b Department of Orthopaedic Surgery, The Johns Hopkins University, Baltimore, MD.

**Keywords:** granuloma, immune response, injection, tumor, vaccine

## Abstract

**Rationale:**

Soft tissue masses are common within the general population with a minority diagnosed as soft tissue neoplasms. Differing between benign and malignant soft tissue processes can be a challenge given the overlapping clinical and imaging characteristics. We present the case of a 69-year-old female referred to the Orthopaedic Oncology Service for evaluation of a suspected soft tissue sarcoma in the upper arm.

**Patient Concerns:**

She reported a mass localized over the deltoid with associated tenderness 1 month after influenza vaccination.

**Diagnosis:**

After thorough consideration of the patient's clinical course, history, advanced imaging, and physical examination, the diagnosis of injection granuloma associated with recent influenza vaccination was considered.

**Interventions:**

Biopsy was deferred and close interval follow-up with clinical and imaging evaluation revealed a resolving process.

**Outcomes:**

The patient was followed until complete resolution of all symptoms, which occurred 5 months after initial presentation.

**Lessons:**

It was hypothesized that due the patient's body habitus, the injection contents intended for intramuscular administration remained in the subcutaneous tissues and elicited a granulomatous reaction. This case highlights several important factors for physicians to consider in the work up of suspicious masses for which injection granuloma is on the differential diagnosis.

## 1 Introduction

Hypodermic administration of vaccines or medications is 1 of the most common medical procedures performed but is not absent of complications. Routine injections of vaccines or medications have been shown to have potential for fat necrosis of the tissues being infiltrated, which may lead to a soft tissue reaction that may either remain asymptomatic or may present with pain and local changes in skin appearance.^[1]^ These reactions are often referred to as injection granulomas, and they most commonly occur in the upper outer quadrant of the soft tissues surrounding the buttocks or deltoid musculature.^[2]^ Injection granulomas are believed to occur more commonly in subcutaneous fat as opposed to skeletal muscle due to the comparatively poorly organized vascular network, which leads to slower absorption of the injected material and therefore greater time for irritant exposure.^[2]^ Females are believed to be more susceptible than males due to sex-based differences in soft tissue envelopes overlying common injection sites.^[3]^

On magnetic resonance imaging (MRI), these lesions may be hyperintense or hypointense on T2-weighted sequences due to variable stages of necrosis, edema, hemorrhage, or fibrosis.^[1]^ Given their widely variable characteristics on MRI, they may mimic malignant processes, such as soft tissue sarcomas. Therefore, these lesions require proper evaluation by a clinician, with special consideration to a thorough and history and physical examination, advanced imaging, and observation for progression or resolution.

## 2 Statement of informed consent

The patient was informed that data concerning this case would be submitted for publication and verbally agreed.

## 3 Case report

**Figure 1:**
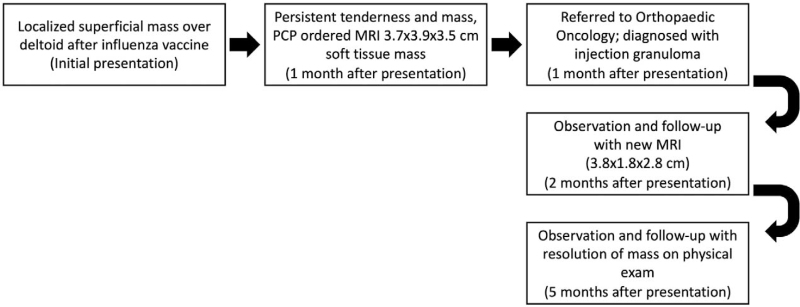
Timeline of patient's symptoms, physical exam and imaging findings, and subsequent management.

A 69-year-old African American female initially presented to her primary care provider for evaluation of a soft tissue mass in her right upper arm, which she noticed after receiving a seasonal influenza vaccine to the area (Fig. 1). At presentation, the patient's past medical history was significant for anti-synthetase syndrome (on Rituximab, complicated by pulmonary hypertension and interstitial lung disease requiring home oxygen), idiopathic angioedema, hypertension, chronic kidney disease, iron deficiency anemia, mixed hyperlipidemia, morbid obesity and obstructive sleep apnea. Her family history was significant for breast cancer in her mother and prostate cancer in her father. Her social history was notable for a remote history of smoking.

**Figure 2:**
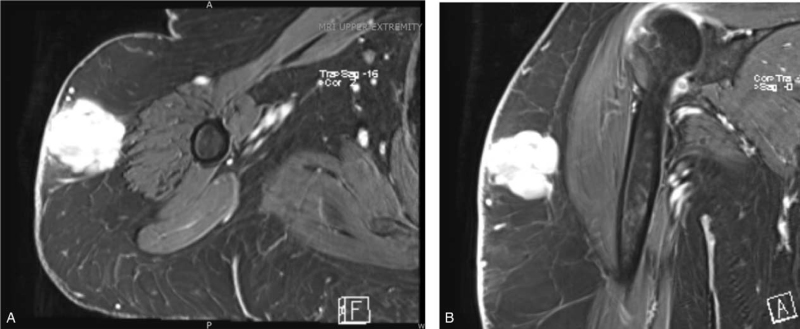
Initial presentation MRI A) axial, volumetric interpolated breath-hold examination (VIBE) sequence with gadolinium contrast; B) coronal VIBE sequence with gadolinium contrast.

**Figure 3:**
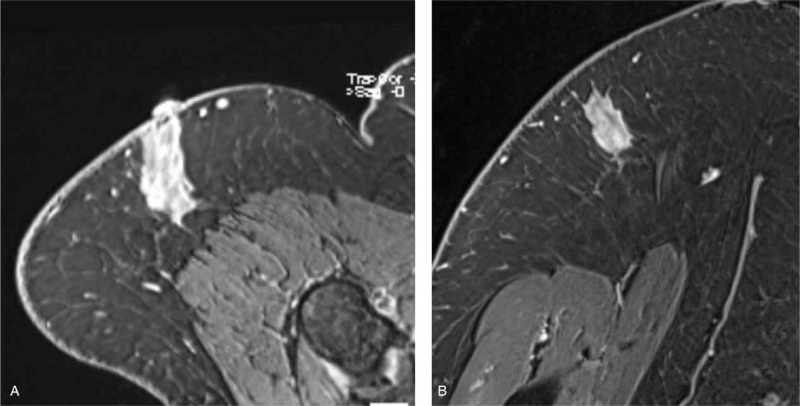
Follow-up MRI, 1 month following initial MRI. A) Axial VIBE sequence with gadolinium contrast; B) coronal VIBE sequence with gadolinium contrast.

The patient's primary care physician obtained an MRI 1 month after initial presentation, which revealed a 3.7 x 3.9 x 3.5- centimeter heterogeneous mass in the soft tissues overlying the deltoid muscle (Fig. 2). Given this finding, she was then referred to the Orthopaedic Oncology Service for further evaluation and management. On presentation, she described a mass in the vicinity of the deltoid injection site. She described vague discomfort in the area, particularly with external compression or direct applied pressure, such as when lying on her shoulder. She noted that the mass had slightly decreased in size since initial presentation. Physical examination revealed a palpable mass over the shoulder which was moderately tender to palpation without overlying skin changes. Her shoulder range of motion was within normal limits as was her upper extremity motor and sensory exam. Due to the reported interval decrease in size, MRI appearance, and the timeline of receiving her influenza vaccine, an injection granuloma was suspected. She was recommended observation with serial examination and underwent a repeat MRI at a 1-month interval, which demonstrated a decrease in size of the mass to 3.8 x 1.8 x 2.8 centimeters (Fig. 3). On examination 3 months later, a mass could not be palpated. She ultimately experienced complete resolution of the mass and did not require any further intervention.

## 4 Discussion

MRI is widely utilized in the evaluation of soft tissue masses. In our patient, the small size, superficial location, and lack of invasion to nearby structures suggested a benign process. Benign etiology was supported by a decrease in size since initial presentation, as well as the timeline of recently receiving a seasonal influenza vaccination.

The Centers for Disease Control and Prevention (CDC) recommend intramuscular (IM) administration of the influenza vaccine to the deltoid.^[4]^ Stronger immune responses have been demonstrated for intramuscular administration compared to subcutaneous administration for a variety of vaccines, including influenza.^[2]^ Furthermore, superficial administration, such as subcutaneous or intradermal injection, causes increased local irritation, inflammation, necrosis, and granuloma formation.^[3]^ The differences in biological response between subcutaneous and intramuscular administration may be due to anatomic differences in vascularity as well as vaccine bioavailability resulting in slower mobilization and processing of antigen and irritants when injected into subcutaneous fat.^[3,5]^

Injection granulomas mimicking sarcoma have previously been descried in the buttocks.^[6]^ In a case series by Michaels et al,^[7]^ one patient underwent injection granuloma excision due to features particularly concerning for soft tissue sarcoma on physical examination. The increased incidence of injection granulomas in the buttocks is likely due to the differing thickness of subcutaneous fat envelope compared to the shoulder, leading to more frequent unintentional subcutaneous administrations.

It has been theorized that injection granulomas occur more commonly in women due to gender-based differences in thickness of soft tissue envelopes overlying common injection sites.^[3]^

This case aligns closely with previously described reports of injection granulomas in other anatomic locations. Notably, this case presents a soft tissue mass of the shoulder occurring in a woman following vaccine administration, with interval decrease in size over time. This patient's injection granuloma occurred in the upper arm, a reportedly less common location. We hypothesize that the body mass index (BMI) of 44 kg/m^2^ in our patient contributed to failed intramuscular localization of the vaccine due to increased adipose tissue thickness over the injection site. This hypothesis has been previously supported for gluteal IM injections, which fail using standard needle lengths for women with BMI greater than 30 kg/m^2^ and men with BMI greater than 35 kg/m.^[2,4]^

This case further demonstrates that BMI needs to be considered when planning IM injections, as inadvertent subcutaneous injection is not limited to the buttocks. It is not known whether the patients complex past medical history contributed to the robust inflammatory reaction observed. Furthermore, IM injection of the influenza vaccine is preferred to ensure adequate immune response as recommended by the CDC. Adherence to CDC guidelines regarding needle length may decrease the incidence of inadvertent subcutaneous injection. Another possible solution may be to estimate subcutaneous fat thickness based on cross-sectional imaging to help determine appropriate needle lengths.

There are several limitations of this study. First, this is a single case report reviewed retrospectively from the electronic record. A second limitation is our diagnosis was not confirmed with a tissue sample via biopsy. However, we have 5 months of data supporting a benign and resolving process suggestive of our diagnosis being an injection granuloma. A strength of this study is in our reporting of a benign mass, all while recognizing and identifying a wide spectrum of differential diagnoses, in an increasingly critical time for vaccinations. As the number of vaccinations grow in the current Coronavirus era, we need to be cognizant of areas for improvement and the subsequent complications of vaccine injections that may arise.

## 5 Conclusion

In summary, an injection granuloma, secondary to vaccine administration to the subcutaneous fat, may be confused with a soft tissue sarcoma on initial physical examination and MRI. Recognition of the imaging characteristics and location/ progression of the mass will aid in differentiating between an injection granuloma and a malignant process, such as a soft tissue sarcoma.

## Author contributions

**Conceptualization:** Gregory R. Toci, Carol D. Morris.

**Data curation:** Gregory R. Toci, Carol D. Morris.

**Formal analysis:** Gregory R. Toci, Christa L. LiBrizzi, Adam Scott Levin, Carol D. Morris.

**Investigation:** Christa L. LiBrizzi, Jarred A. Bressner.

**Supervision:** Adam Scott Levin, Carol D. Morris.

**Writing - original draft:** Gregory R. Toci, Jarred A. Bressner, Adam Scott Levin, Carol D. Morris.

**Writing - review & editing:** Gregory R. Toci, Christa L. LiBrizzi, Carol D. Morris.
